# Titanium production by magnesium thermal reduction in the electroslag process

**DOI:** 10.1038/s41598-019-54112-2

**Published:** 2019-11-26

**Authors:** Ernests Platacis, Imants Kaldre, Ervīns Blumbergs, Linards Goldšteins, Vera Serga

**Affiliations:** 1 Institute of Physics University of Latvia, Miera 32, Salaspils, LV-2169 Latvia; 2 Institute of Inorganic Chemistry, Riga Technical University, P. Valdena 3/7, Riga, LV-1048 Latvia

**Keywords:** Applied physics, Chemical engineering

## Abstract

Titanium is widely used in specific applications due to its high strength, low density and good chemical stability. Despite it is one of the most abundant elements in the earth’s crust, it is very expensive, because production of pure metallic titanium is very complex. Kroll process is the way how most of the titanium is produced nowadays. Shortages of this process are that it is batch process and it is very energy exhaustive, because titanium sponge material after reduction reaction needs complex post processing to isolate pure titanium. In this work we describe and experimentally investigate technology for Ti production from titanium tetrachloride using combined Kroll and electroslag process. Such process allows to achieve better reaction product separation by molten slag and process can potentially be continuous, thus technological process to produce metallic titanium can be significantly shortened.

## Introduction

Titanium and its alloys have extraordinary mechanical properties and low density, which makes them important materials in aerospace and other industries^1^. Complexity and high energy consumption of the titanium production by Kroll process is one of the limiting factors for wider application of titanium and its alloys. Main raw material for titanium production is rutile ore (mainly TiO_2_). It is treated with chlorine producing titanium tetrachloride (TiCl_4_), which is then reduced by magnesium at a temperature of 800–900 °C. Reduction reaction takes place and Ti and MgCl_2_ sponge is formed.

$${{\rm{TiCl}}}_{4}+2{\rm{Mg}}=\, > {\rm{Ti}}+2{{\rm{MgCl}}}_{2}$$

After Kroll process material requires several thermal post-treatment steps to isolate pure titanium. In industrial scale Ti production is done in confined stainless-steel reactors of several cubic meter volume. Ti reduction reaction is exothermic, releasing 412 kJ/mol or 686 kJ/mol if magnesium enters in reaction in gaseous state^2^. After loading the components reactor must be maintained at high temperature for several days while reaction takes place. Then reactor is cooled down and cut-open. Material in the reactor (titanium sponge) contains small Ti droplets, reaction products and remains of unreacted components. Ti sponge from reactor has different quality regions, which then are grinded and sorted, and undergoes different postprocessing stages^3,4^. It takes several vacuum arc remeltings to isolate high purity metallic titanium from titanium sponge. Kroll process technology is well established and widely used in industry, however not all process steps are fully understood^5,6^. More effective production technology is searched in various directions. Powder metallurgy of titanium is one of the ways to reduce cost of titanium extraction from Ti sponge. Powder metallurgy is used in various applications, but material quality is lower^7^. Idea of improved Ti and reaction product separation in liquid salt environment has been described^8^. Chemical and metallothermic reduction possibilities to produce Ti and its alloys have been described by some authors^9–12^. Recently hydrometallurgy processes are developed to upgrade rutile quality by leaching of ilmenite FeTiO_3_^13^. Nowadays available titanium ore quality is decreasing and typically rutile content can be as low as 1%.

Electroslag remelting (ESR) has been used in various industrial metallurgy processes. It is one of the refining procedures for various metals, allowing to produce ingot with precise composition and properties^14,15^. ESR allows to achieve high temperature and to regulate process parameters easily, hence it is also widely used for titanium purification^16^. Electric current heating of the titanium reduction reactor has been studied recently, however the feasibility of the process have not yet been demonstrated due to the technical complexity of the task^17^. Accurate numerical simulations of the ESR process are complicated because of many physical effects which must be considered, and difficulties to precisely determine physical properties. Nevertheless, various numerical models have been developed, analysing electric heating, liquid slag flow, inclusion distribution and other physical processes. It is shown that main slag flow is caused by natural convection due to localized heating near the electrode tip^18,19^. Idea to realize combined Kroll and electroslag process in search on improved technology has been described recently^20^. Previous work demonstrates that electric heating allows to reach high temperature, melt slag and maintain environment for the reduction reaction for long time^21^. This technology is scalable because electric current allows to supply large heat density in the reactors of various sizes. ESR process in large size is described and used in industry^22^.

Purpose of this work is to develop and investigate technology for titanium production in combined Kroll and electroslag process. Process is numerically and experimentally investigated, its feasibility and potential for industrial application and scalability has been assessed. Experiments in small scale reactor is done to identify main problems and to test various experiment conditions and its impact on the reaction outcome.

## Experimental

Main part of the experimental setup is water-cooled cylindrical stainless-steel reactor. Reaction products and slag are injected in the reactor and heated by electric current. The dimensions of the reactor make it possible to study the process of reduction of titanium from titanium tetrachloride with Mg as a reducing agent. Reactor size allows to inject components to obtain 5 moles of titanium (240 g) without removing the reaction products. This allows to investigate multiple component injection. Numerically calculated temperature distribution in the reactor indicates the reactor size and optimal aspect ratio allowing to achieve reaction temperature with available power supply.

**Figure 1:**
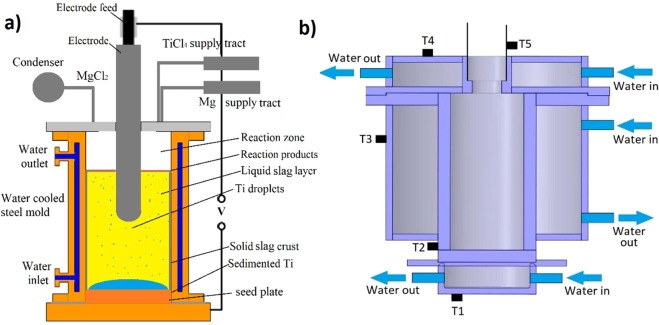
(**a**) Principal scheme of the experiment; (**b**) Cross section of the water-cooled stainless-steel reactor (T-thermocouples).

Reactor is designed with possibility to be vacuumed or filled with argon. Experimental setup is equipped with supply systems for reaction components, electrode feed mechanism and exhaust, and condenser for reaction products. Current can be applied between electrode and bottom seed plate. Experimental setup allows to investigate reaction kinetics in liquid and gaseous phases and test different slag mixtures which can be used for separation of titanium and reaction products. Experimental setup allows to use two electrodes, compound electrode and different seed plate materials. Methods how to inject Mg and TiCl_4_ has been analysed and technical difficulties have been tackled by developing magnesium and TiCl_4_ dosing systems and supply tracts. Schematics of the experiment is shown in Fig. 1a. Reactor chamber inner diameter is 60 mm and height is 160 mm. Reactor cross section is shown in Fig. 1b. Separate water cooling for reactor walls, lid and bottom are made to enable various temperature regimes in the reactor. Temperature during the experiment is monitored by several thermocouples as shown in Fig. 1b. Temperature measurements on the outer walls allows to make comparison to numerical models and to estimate reactor inner temperature.

**Figure 2:**
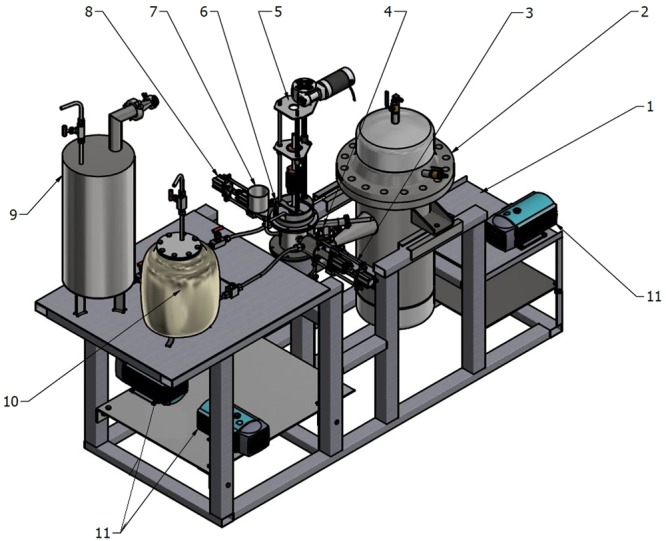
Assembly of the experimental setup: 1-Stainless steel frame, 2-MgCl_2_ condenser, 3-Liquid magnesium dispenser, 4-Exhaust valve, 5-Electrode feed mechanism, 6-Reactor, 7-Reactor filling cone, 8-TiCl_4_ dispenser, 9-TiCl_4_ tank, 10-Magnesium tank, 11-Vacuum pumps.

Experimental assembly is shown in Fig. 2. TiCl_4_ and Mg is supplied via specially designed dispensing systems (3,8), which allows precise dosing of each component at desired temperature. Slag can be either filled into reactor as solid powder (dry start) or poured into pre-heated reactor via filling cone (liquid start) (7). Gaseous and liquid reaction side products (MgCl_2_) can be periodically evacuated from the reactor and collected in condenser (2). Mg excess should be maintained in the reactor to ensure maximum reduction reaction efficiency. Cu, Al and stainless-steel seed plates can be used at the reactor bottom. Argon can be supplied to the reactor at a rate of 4–10 l/min. To start the process, electrode is placed close to the bottom of the reactor and voltage is applied. Distance is then gradually increased until stationary regime is achieved. For stable start of the electroslag process during the experiments, the gap between the tip of the electrode and seed plate is gradually changed from 0 to 30 mm.

To obtain information on the processes occurring during the conducted experiments, slag and reaction product samples were taken from different zones of the reactor after the reactions. The approximate content of elements was established using semi-quantitative X-ray fluorescence (XRF) analysis. The phase composition of the samples was studied using X-ray diffraction (XRD) analysis. Scanning electron microscopy (SEM) and optical microscopy were used to visually observe material morphology.

### Analytical description

Estimate of characteristic dimensionless numbers for typical experiment is done to identify main physical effects which have to be considered in numerical models and during interpretation of the experimental results. NaCl physical properties from Table 1 are used in following estimations and numerical models. For typical experiment, measured electrical parameters are *U* = 33 V, *I* = 300 A, and distance between electrode and seed plate at steady regime is *L* = 30 mm. Total electrical power dissipated in the reactor is *P* = *I* *·* *U* = 10 kW. Typical slag mass in the reactor is up to m = 500 g. Time to heat slag from *T*_0_ = 300 K to *T* = 1300 K and melt it can be calculated according to Eq. 1. In experiments heating and melting takes about 5 minutes due to heat flow to the walls, reactor own heat capacity and unsteady electric power.

1 $$t=\frac{c\cdot m\cdot (T-{T}_{0})}{P}+m\cdot k=90\,\sec $$

**Table 1:** Physical properties of the NaCl slag and electrode, and reactor materials.

	NaCl (solid)	NaCl (liquid) at T_m_	Symbol	Tungsten	AISI304 steel	Unit
Density	2100	1460	ρ	18000	7900	kg/m3
Heat capacity	1690	1280	c	130	480	J/kg·K
Thermal conductivity	1.0	0.1	λ	173	15	W/m·K
Electric conductivity	50	100	σ	1.5·107	1·106	Sim/m
Viscosity		0.005	μ			Pa·s
Surface tension		0.285	γ			N/m
Heat of fusion	5.5·105		k			J/kg
Thermal expansion coefficient		3·10–4	β			1/K

Buoyant versus viscous force ratio is characterized by Grasshof number. Characteristic temperature difference within the reactor is assumed to be *∆T* = 200 K. From Eq. 2 we get Gr = 11, which shows that buoyancy is the main force driving liquid flow.

2 $$Gr=\frac{g\rho \beta \Delta T{L}^{3}}{\mu }=11$$

Fully molten slag flow velocity can be estimated by balancing Buoyancy and inertial forces in simplified Navier-Stokes equation. Equation 3. gives velocity of u = 2 cm/s.

3 $$\beta \rho g\Delta T=\frac{\rho {u}^{2}}{L}$$

The estimated Peclet number, which characterizes ratio between convective and diffusive heat transfer is given by Eq. 4. Pe = 600 means that convective heat transfer is dominant and heat conduction in liquid slag is much smaller.

4 $$Pe=\frac{\rho Rv{C}_{p}}{k}=600$$

Reynolds number describes the relationship between fluid inertia and viscous forces and determines the type of fluid movement. If characteristic velocity is assumed to be 0.02 m/s, Reynolds number is 180 according to Eq. 5, thus liquid slag flow is laminar.

5 $$Re=\frac{\rho Rv}{\mu }=180$$

Electrovortical flow is created by interaction between electrode current and magnetic field generated by electrode current^23^. This phenomenon is known in welding and electric arc processes where high electric current density is passing through the liquid conductive media. In given case electrocortical force can be estimated according to Eq. 6. We get force density f = 500 N/m^3^ which is small compared to other forces (gravity, buoyancy).

6 $$f=j\cdot B=\frac{I}{\pi {R}^{2}}(\frac{{\mu }_{0}I}{2\pi R})=\frac{{\mu }_{0}{I}^{2}}{2{\pi }^{2}{R}^{3}}$$

### Numerical results

**Figure 3:**
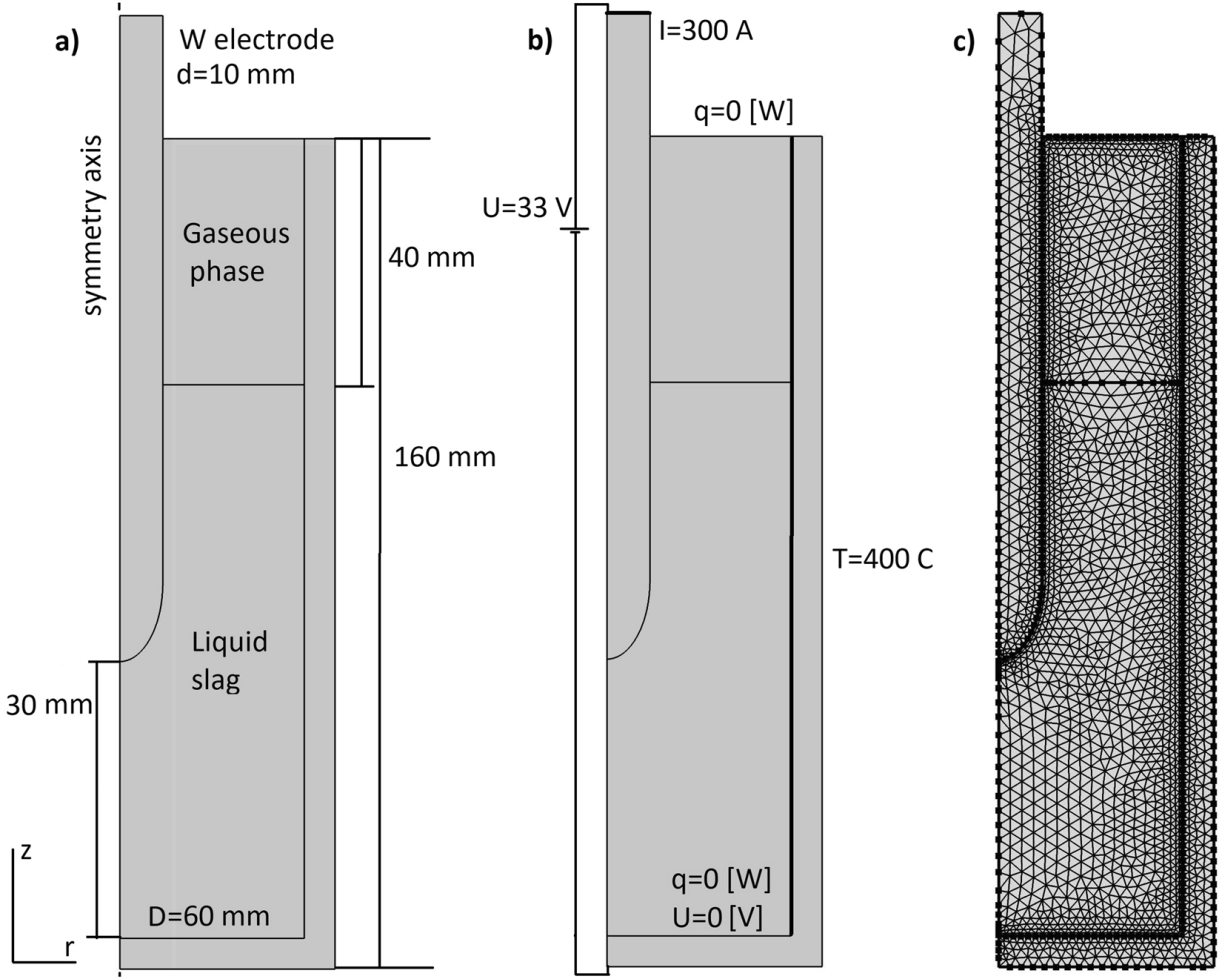
(**a**) Geometry of the numerical model; (**b**) Boundary conditions for electrical and heat transfer problems; (**b**) Mesh of the model (5000 elements).

Numerical model to calculate temperature distribution in the reactor, buoyancy convection velocity and its influence on the temperature distribution is developed. Axisymmetric model is made for electromagnetic, thermal, fluid flow and phase change calculations. Model geometry and domains are shown in Fig. 3a. Fluid dynamics is calculated only in 120 mm high liquid domain. Figure 3b shows thermal and electric boundary conditions. No-slip boundary condition and laminar model is used for fluid flow model. Figure 3c shows tetrahedral mesh of 5000 elements used in numerical simulations. Numerical model parameters are chosen based on measured values during typical experiment (*I* = 300 A, *U*=33 V). Electric current is injected into molten NaCl slag through 10 mm diameter tungsten electrode and negative terminal is the bottom seed plate of the reactor. Reactor inner side walls are assumed to be electrically insulating because they are covered with insulating solid slag crust during experiment. Side walls are water cooled, thus inner wall is assumed to be isothermal at 400 K according to thermocouple measurements. Bottom and lid are not water cooled to ensure that slag is molten from bottom to top, and heat losses to cooling water are not too high.

**Figure 4:**
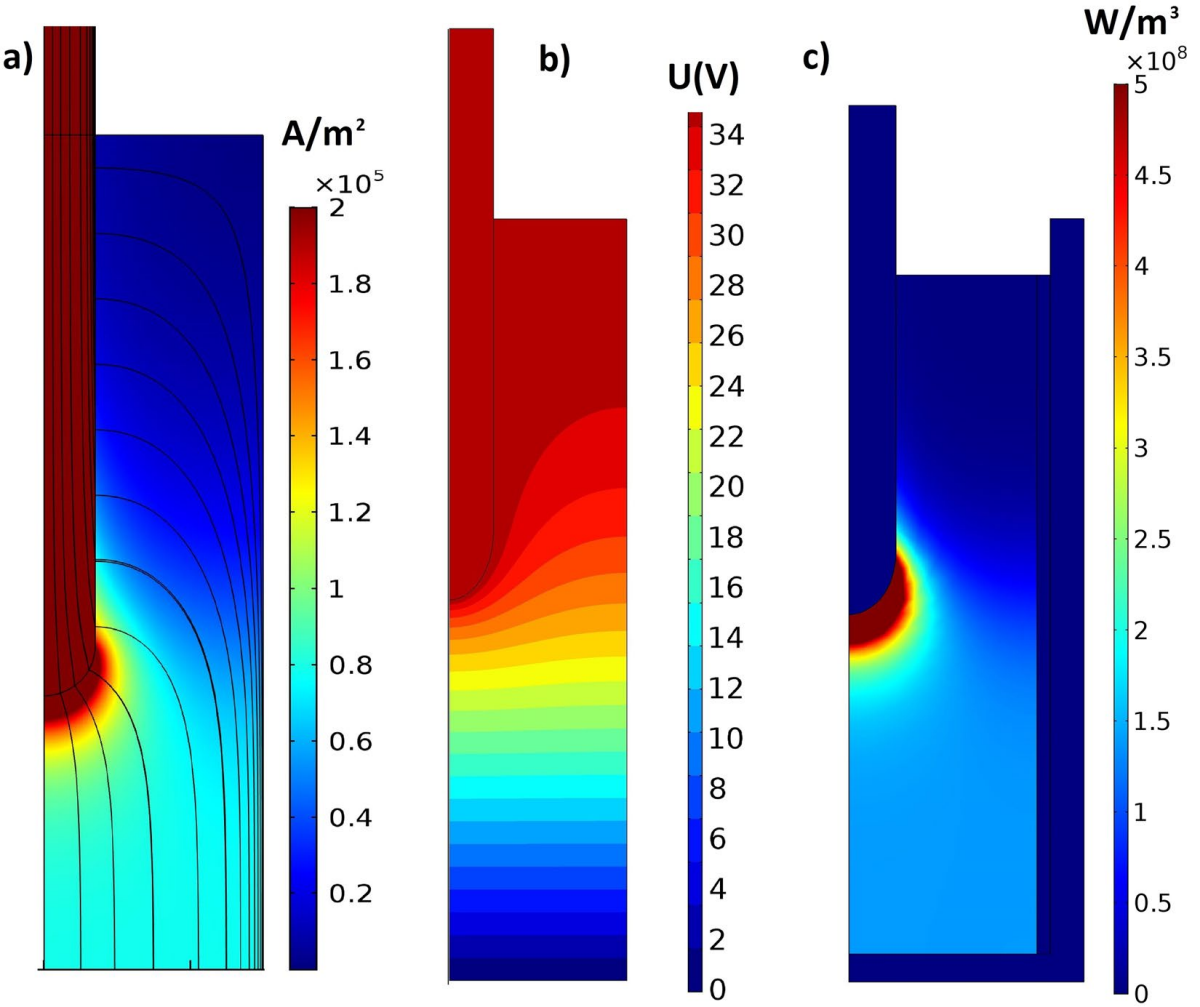
Electric calculations of electroslag process. (**a**) Numerically calculated electric current density. (**b**) Electric potential; (**c**) Joule heat density.

Numerically calculated electric current density is shown in Fig. 4a, electric potential distribution is shown in Fig. 4b, and Joule heat density is shown in Fig. 4c. Due to the large difference in electrical conductivities between slag and electrode, all the heat is released in the slag near the tip of the electrode.

**Figure 5:**
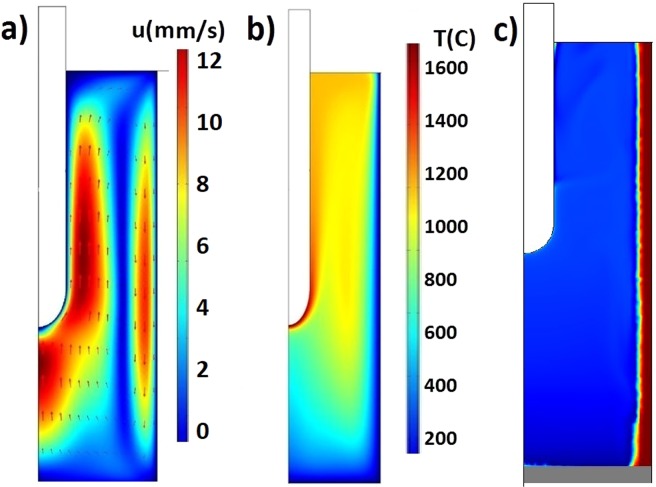
Numerical calculation results. (**a**) Molten NaCl slag velocity due to buoyancy convection. (**b**) Temperature distribution in the reactor. (**c**) Solid slag crust on the inner wall.

Near the tip of the electrode slag is overheating and significant liquid slag convection appears if slag is molten. Hot slag is rising near the electrode and sinks back along the water-cooled outer wall. This convection is the main heat transfer mechanism in the reactor. Multiphysical model is created to calculate slag flow and its impact on the temperature distribution in the reactor. Model considers gravity and linear density-temperature dependence 2061–0.476·T[K]^24^. Calculated liquid slag convection velocity is shown in Fig. 5a. Calculated slag velocity magnitude up to 12 mm/s is in good agreement with analytically estimated value. Temperature distribution in reactor is shown in Fig. 5b, showing that temperature near the electrode tip reaches 1600 °C. Temperature distribution is significantly altered by liquid slag flow. Numerically calculated solid slag crust on the water-cooled reactor wall is shown in Fig. 5c. Using steady state phase change model, we calculate solid slag crust on the side wall of the reactor. This 3–5 mm crust plays an important role by protecting reactor walls from erosion and reducing heat losses, because solid slag has very low thermal and electrical conductivities.

Numerical calculations and analytical order of magnitude estimates provide us with information about the expected temperature distribution and possible liquid motion in the reactor. Numerical model indicates the thickness of the wall crust during the experiment. If slag is fully molten, then convective heat transfer will significantly affect the temperature distribution in the reactor, avoiding overheating near the electrode tip. Numerical model and analytical estimates show that planned electrical parameters is enough to achieve needed temperature.

## Experimental Results And Discussion

The implementation of the Kroll process combined with the electroslag process in our proposed reactor design and verification of the theoretical and numerical calculations requires experimental studies, which are summarized in this section. Experiment series are done to determine the impact on various parameters and to identify main technical problems. Experiments are described in sequence starting with simpler experiments to test electrode melting and slag behaviour to more complex experiments with Mg and TiCl_4_ injection and analysis of post-reaction material. Conditions of the most significant experiments are summarized in Table 2.

**Table 2:** Summary of conditions of the experiments.

Exp. No	Slag			Electrode	Seed plate	Distance between electrode and seed plate, mm	Technical conditions		
	Slag mass, g	Composition	Initial state				I, A	U, V	t, min
1	500	NaF	liquid	Ti	^**^Cu	3–20	400	25	30
2	715	67wt.%NaF + 3 wt%NaCl + 30wt.%Al_2_O_3_	liquid, solid	Ti	^**^stainless steel	3–20	400	25	20
3	500	NaCl	liquid	Ti	^**^Al	12–17	360	12–15	100
4	500	NaCl	liquid	Ti	^**^Cu	0–20	500	8–20	30
5	350	89wt.%NaCl + 11wt.%CaF_2_	liquid, solid	Ti	^**^stainless steel	12–30	200	9–13	90
6	280	NaCl	liquid	Composite stainless steel-Mg	^**^stainless steel	3	400	13	40
7	500	NaCl	liquid	Combined W-Mg	^*^Cu	5	400	12	60
8	500	NaCl	liquid	Combined W-Mg	^**^stainless steel	5	300–400	13	20

^*^Water cooled seed **No seed cooling.

Experimental studies were started with studying the process of electroslag remelting of a titanium electrode to investigate Ti droplet filtration through the slag layer as electrode is gradually melting. The need for this experiment is to investigate the possibility of melting the titanium sponge, which is formed in Kroll reaction. Sponge is in liquid state in the liquid slag environment which ensures separation of titanium and reaction products due to their different densities. The choice of slag composition in the experiments No. 1 and No. 2 was determined by the possibility of forming a dense protective layer on the inner surface of the reactor - a crust layer to prevent the formation of a FeTi eutectic (T_melt_ = 850–900° С), which could lead to damage of the reactor walls and pollution of the reaction products. During experiments, the necessary electrical parameters of the processes were determined to achieve melting temperature of the titanium (1670 °С). After experiments dense slag crust on the walls is observed. The results of X-ray fluorescence analysis of samples of a slag crust after the experiments are presented in Table 3.

**Table 3:** Results of X-ray fluorescence analysis of crust samples at the reactor walls from the experiments No. 1 and No. 2.

Exp. No	Chemical composition, wt%										
	NaF matrix	Ti	Ca	Fe	Cu	Al	Cl	Cr	Ni	Mo	Mn
1	99.8	0.06	0.04	0.02	0.02	−	−	−	−	−	−
2	69.8	8.3	0.04	0.68	0.02	17.6	3.1	0.17	0.10	0.04	0.02

According to the XRD data, when using one-component slag-NaF (Exp. No.1), the crust sample does not contain impurity phases. Presence of Al_2_O_3_ additive in slag (Exp. No. 2) leads to the formation of sodium hexafluoroaluminate and sodium aluminate (Fig. 6.2). Thus these data are in good agreement with the results of X-ray fluorescence analysis (Table 3). XRD analysis of samples from the surface of a metallic ingot obtained on a stainless-steel seed plate (Exp. No. 2) showed that a solid solution of oxygen in titanium Ti_6_O is present in the sample (Fig. 6.1). The SEM image of the remelted metal is shown in Fig. 7a. Optical microscope image Fig. 7b shows that sample is metallic with slag inclusions. Thus, from sample analysis we may conclude that titanium has been melted and sedimented on the seed.

**Figure 6:**
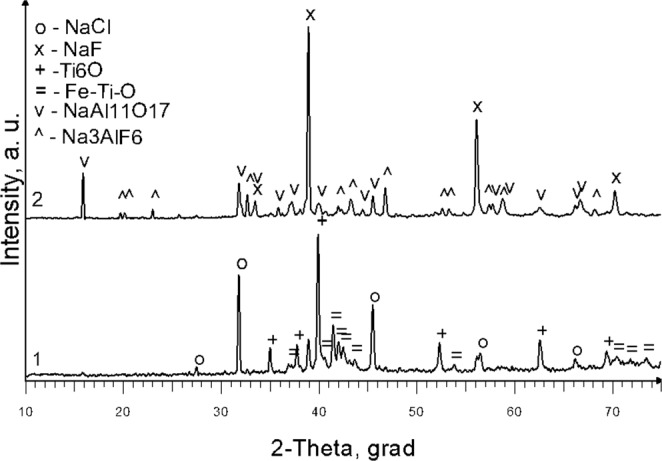
X-ray diffraction spectrum: 1- sample from the surface of the ingot on the seed (Exp. No. 2); 2- solid slag crust (Exp. No. 2).

**Figure 7:**
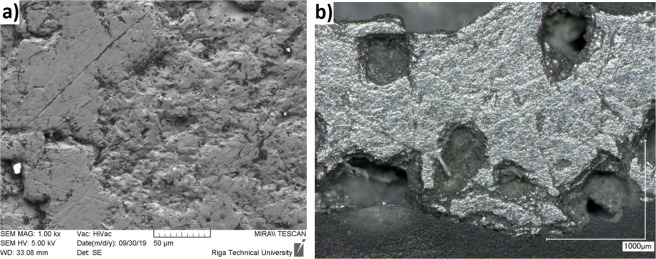
Surface microstructure of the remelted metal: (**a**) SEM image; (**b**) Optical micrograph of remelted titanium ingot (Exp. No. 2).

XRF analysis results of the metallic material deposited on the seed (Table 4) shows the presence of significant amounts of impurity elements compared to initial electrode material. In case of Exp. No. 1 copper seed is dissolved. Iron could be present as a result of crucible wall decay due to high electric power in these experiments (10 kW).

**Table 4:** Results of X-ray fluorescence analysis of the electrode and metal samples from the seed plate (Exp. No. 2).

No Exp.	Chemical composition wt.%	Ti	Cu	Zn	Fe	Ca	Si	Al	NaF	Cr
1,2	Electrode initial material	97.2	1.3	0.76	0.48	0.08	0.09	0.09	−	−
1	Metal on the surface of the seed	70.8	9.4	2.7	10.4	0.39	0.51	0.33	5.4	−
2		43.6	0.09	−	7.0	−	0.64	5.5	39.9	1.7

**Figure 8:**
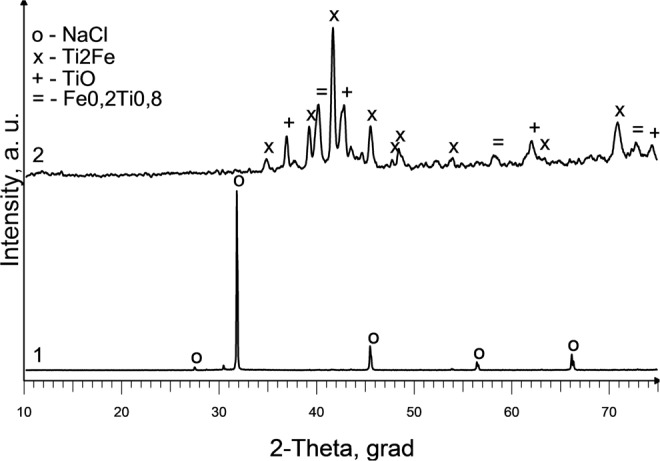
X-ray diffraction spectrum of samples from crust over the slag bath (Exp. No. 3): 1- Sample before leaching; 2 - after leaching with water.

It is known that TiCl_4_ has high solubility in a NaCl melt (165 gmol/m^3^ at 821 °C^25^). Therefore, to study the reduction reaction of titanium tetrachloride with magnesium in the molten slag environment sodium chloride (T_m_ = 800.8 °C) was chosen as main slag component. Series of test experiments was carried out without introducing the components for the Kroll reaction (Exp. No. 3–5). After the completion of these experiments, the formation of a slag crust layer on the walls of the reactor was detected. The thickness of the crust is from 2 to 4 mm, which agrees well with numerical calculation showed in Fig. 5(c). To ensure better deposition of reduced titanium during the Kroll process, materials with different thermal conductivities were used as seeds. In experiments No. 3–5, Al, Cu and stainless steel, were used as seeds. Experimental results show that the best material for seed is stainless steel. Copper and aluminium have a negative effect on the electrical parameters, and they are more soluble in hot slag than stainless steel. Results of the XRD phase analysis of the crust material collected from the inner walls of the reactor above the slag bath in experiment No. 3 is shown in Fig. 8. Impurities identified in the composition of the slag are the components from reactor material-alloy steel.

**Figure 9:**
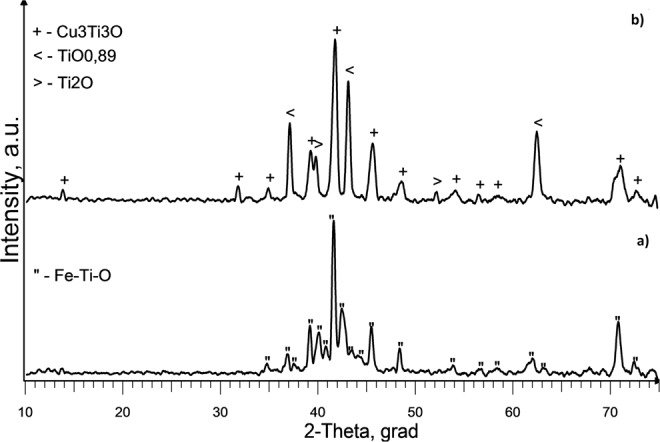
X-ray diffraction spectrum of samples from the lower part of the slag bath after leaching with water: (**a**) Exp. No. 3; (**b**) Exp. No. 4.

XRD analysis of slag samples from the reactor lid and from the surface crust located above the slag bath showed the presence of only one crystalline phase-NaCl. These results indicate that the slag is heated above its boiling point of NaCl (1465 °C). The presence of small amounts of titanium in the composition of the slag located in the lower part of the slag bath (between the end of the electrode and the seed) is confirmed by XRF analysis (Table 5). After leaching of these slag samples with water, the following crystalline phases were detected: Fe_2_Ti_4_O, FeTiO, Ti_9_Fe_3_O_3_ (Exp. No. 3) and TiO_0.89_, Ti_2_O, Cu_3_Ti_3_O (Exp. No. 4). The presence of these impurities is likely due to erosion of the metal elements of the reactor during the process. Corresponding XRD spectrums are shown in Fig. 9a (Exp. No.3) and Fig. 9b (Exp. No. 4). Thus, it may be concluded that in these experiments melting temperature of Ti was not exceeded.

**Table 5:** Results of X-ray fluorescence analysis of slag samples located between the end of the electrode and the seed (Exp. No. 3 (seed Al) and Exp. No. 4 (seed Cu)

Exp. No	Chemical composition, wt%								
	Cl	Na	Ti	Fe	Cr	Ni	Mo	Mn	Cu
3	47.5	29.2	2.5	0.84	0.22	0.12	0.05	0.03	0.03
4	56.5	38.3	3.1	0.59	0.13	0.08	0.02	−	0.27

**Figure 10:**
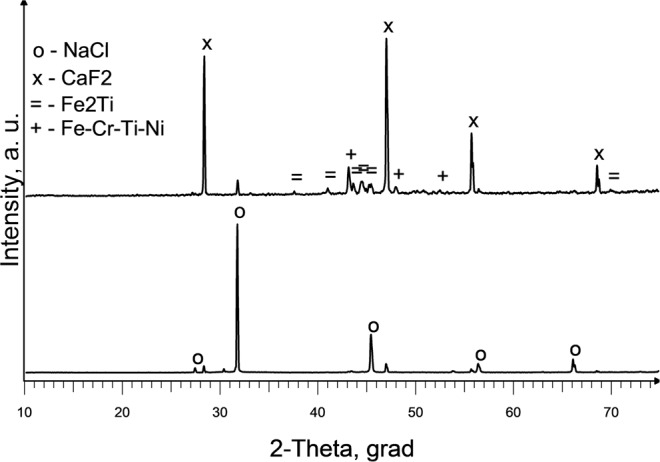
X-ray diffraction spectrum of slag samples from the seed (stainless steel) surface (Exp. No. 5): 1 –before and 2- after leaching with water.

Calcium fluoride is widely used as slag in the production of high-quality metals^26^. In the experiment No. 5 CaF_2_was used as an additive to the main component of the slag - NaCl. With multicomponent slag we can regulate reactor temperature, because of different electrical conductivity of the slag. After completion of the experiment, weighing of the titanium electrode is done and it is found that the mass decreased by 4 g. However, elemental titanium was not found on the seed surface. There are small amounts of intermetallic compounds-alloy steel phases in slag sample taken from the seed (FeCrTiNi, Fe_2_Ti) (Fig. 10)

According to the results of XRF analysis, titanium content in slag samples from the seed surface and in the volume of liquid slag bath is about 1 wt%. The largest amount of titanium is present in slag samples from the surface of titanium electrode (from ∑ 3 wt% to ∑ 9 wt%). Therefore, the electrode mass loss in this experiment No. 5. is probably attributed to electric erosion of the electrode.

Experiments No. 6 and No. 7 were conducted to study the effectiveness of reducing agent supply system and other possibilities to introduce magnesium in the reactor (melting Mg electrode). The developed liquid magnesium supply system (Fig. 2) consists of a tank for heating and melting metal with systems for providing exact dose of oxide-free melt heated to a temperature exceeding melting point of magnesium at 50 °C. This system is complex, and it was problematic to precisely dose magnesium, thus for test experiments No. 6 and No. 7 alternative solution were used. Solid magnesium electrode feed system with a high-temperature seal. Composite electrode made of stainless steel with Mg tip were used (Exp. No. 6).

During experiment it was observed that melting of magnesium takes place at high speed. Results of electrode weighing before and after the electroslag process showed that the amount of molten Mg corresponds to the calculated amount (∑ 24 g). Distribution of Mg into the slag system was studied. It was found that most of the metal was in the form of individual spherical particles with a diameter up to few milimeters. This result shows that magnesium input can be also done by melting Mg electrode.

**Figure 11:**
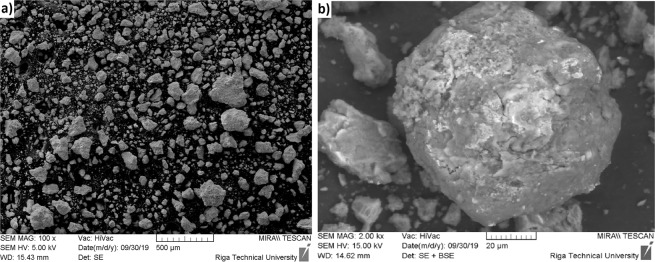
SEM images of Mg particles separated from a slag samples by leaching with water (Exp. No. 7).

**Figure 12:**
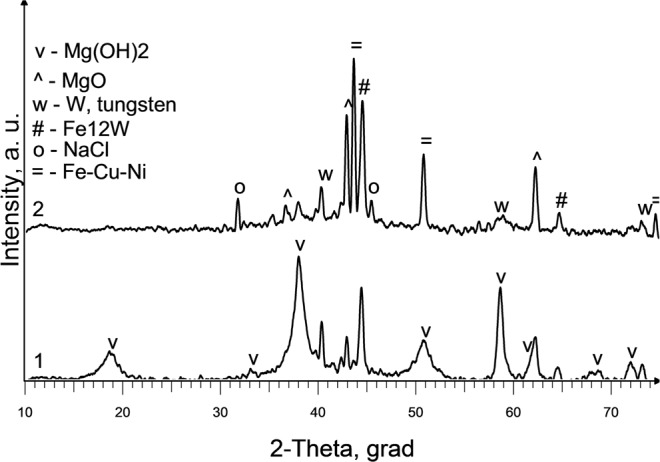
XRD spectrum of slag samples: 1- upper part of the slag bath (layer thickness ∑ 3 cm); after leaching with water; 2 - lower part of the slag bath at the seed surface (layer thickness ∑ 1.5 cm) after partial leaching with water. (Exp. No. 7).

In experiment No. 7 combined W-Mg electrode was used to introduce the calculated amount of liquid magnesium and maintain the slag bath in the liquid state for one hour. An increased duration of the experiment leads an increase in the dispersity of Mg particles distributed in the slag (Fig. 11). According to XRD analysis, the phase composition of impurities in a slag samples from upper, middle and lower part of slag bath is identical (Fig. 12): Mg, Mg(OH)_2_, MgO, W, Fe_12_W, Fe_0.33_Cu_0.33_Ni_0.33_. The composition of impurities in the slag located directly at the boundary of the slag melt - metal is characterized by the presence of a copper seed phase - intermetallic compound Fe_0.33_Cu_0.33_Ni_0.33_ (Fig. 12.1).

Comparative analysis of the proposed X-ray diffraction patterns shows that as the distance from the seed surface increases, the intensity of the diffraction peaks of this crystalline phase decreases (Fig. 12.2). Elements that make up this alloy are enclosed in all slag samples (Table 6). Moreover Mg (OH)_2_ and MgO are formed during leaching as a result of the chemical interaction of magnesium with water. According to X-ray fluorescence analysis results (Table 6) the largest amount of Mg is in the middle part of the slag bath. Presence of the W in a slag composition is probably due to its overheating and slight electrode decay. Thus, X-ray diffraction and X-ray fluorescence analysis from different parts of the reactor allow us to conclude that slag in the reactor is quite well mixed if process is maintained for longer time. Rather high liquid slag convection was predicted by numerical simulation shown in Fig. 5a.

**Table 6:** Results of X-ray fluorescence analysis of slag samples in experiment No. 7.

Location	Chemical composition, wt%								
	NaCl matrix	Fe	W	Cu	Cr	Ni	Mo	Zn	Mg
Upper part of a slag bath	99.5	0.19	0.07	0.06	0.03	0.03	0.01	0.007	−
Middle (main) part of the slag bath	99.6	0.09	0.02	0.02	0.02	0.01	0.01	−	0.12
Lower part of the slag bath	99.5	0.22	0.05	0.09	0.04	0.03	0.01	0.007	0.03

**Figure 13:**
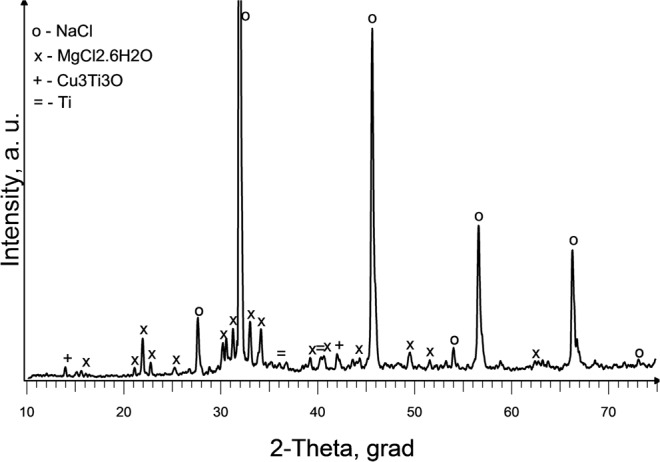
X-ray diffraction spectrum of sample from upper part of slag bath (2/3 of the height) (Exp. No. 8).

Experiment No. 8 with the supply of liquid titanium tetrachloride (170 g) and magnesium (90 g) into the reactor was done to study if reduction reaction takes place and how the reaction products are distributed in the reactor after the experiment. Reaction products were injected in molten NaCl and electric current were maintained for 20 minutes. The results of XRD analysis of material after reaction confirmed the reduction reaction showing the presence of MgCl_2_ and pure Ti (Fig. 13).

However, during dismantling of the reactor and sampling of the material intense smoke emission was noticed. It can be associated with the presence of unreacted titanium tetrachloride, which indicates an incomplete reaction. Thus, component mixing in the reactor may be incomplete or in some parts of the reactor temperature was too low for optimal reaction. According to the results of XRF analysis of slag samples taken from different zones of the reactor (Table 7), titanium content varies from ∑2 to ∑13 wt.%. In metallic droplets found in the slag bath volume near the electrode tip where temperature was highest, titanium content is about 50 wt.%. XRF analysis of a metallic sediment from seed plate shows high magnesium content which indicates that in this zone temperature were too low Titanium content in slag bath metallic sediment is much higher reaching 45.2%. Experiment No. 8 demonstrates that it is possible to obtain metallic titanium deposition using Kroll and electroslag process, however efficiently of this experiment is rather low. This experimental result demonstrates that it is important to maintain high temperature in as large volume fraction as possible. In this experiment it was possible to maintain sufficiently high temperature only in small zone between electrode and seed plate. This problem could be solved using larger reactor where cold near-wall zone will be relatively smaller. Larger reactor with multiple electrodes and additional heating could be a possibility to improve Ti sedimentation on the seed plate.

**Table 7:** Results of X-ray fluorescence analysis of samples in experiment No. 8.

Location		Chemical composition, wt%									
		Cl	Na	Ti	Mg	W	Cu	Fe	Zn	Ni	Mn
Crust of a slag bath (side walls)	Top (2/3 of the height)	70.1	26.5	2.2	0.01	−	0.31	0.14	0.08	−	−
	Lower part (1/3 of the height)	66.3	26.9	2.9	0.76	0.01	1.5	0.49	0.51	−	−
Slag from the slag bath	Top (2/3 of the height)	61.7	19.0	13.0	3.1	−	1.4	0.39	0.88	−	−
	Lower part (1/3 of the height)	67.8	20.7	3.9	2.6	0.10	2.4	0.81	0.82	−	−
	Seed Layer	63.6	23.6	7.6	0.02	−	2.4	1.2	1.0	0.13	−
Sample from the metal surface on the seed		12.9	−	3.5	67.0	−	4.5	1.6	7.2	0.16	1.4
Slag bath metallic sediment		2.4	−	45.2	47.6	−	1.4	0.21	2.4	0.05	0.66
Metallic sediment from W electrode		2.1	−	50.4	40.3	−	2.4	0.4	3.8	−	0.21

## Conclusions

Series of experiments have been carried out for the implementation of the combined Kroll and electroslag processes. It has been experimentally demonstrated that liquid slag may act as a membrane to separate metallic titanium from reaction products after TiCl_4_ reduction with Mg. In this work metallic titanium is obtained and isolated in one step process. Relatively pure metallic titanium was obtained in some parts of the reactor in experiment No. 8. Titanium reduction reaction were experimentally verified in small scale experiment, showing that reaction takes place and metallic Ti distribution and can be altered by varying process parameters like electric parameters, electrode materials and slag composition. Tungsten electrode and stainless-steel seed are the best choice for process using NaCl based slag.

In further studies, to improve the purity of titanium, it is planned to use slags with boiling point above the melting point of titanium. This could prevent slag boiling and evaporation. In further studies it is planned to do more detailed experimental series to determine optimal electric and component injection regime. Larger reactor should be used because it is difficult to maintain high temperature in this small-scale reactor.
